# The Elderly and the City: Lack of Knowledge on Violence Perception and Consequences on Daily Life

**DOI:** 10.2174/1745017901814010046

**Published:** 2018-02-28

**Authors:** Alessia Bramanti, Ernesto D’Aloja, Federico Cabras, Pasquale Paribello, Maria Francesca Moro, Jutta Lindert, Mauro Giovanni Carta

**Affiliations:** 1 Istituto di Scienze Applicate e Sistemi Intelligenti, ISASI, Messina, Italy; 2 IRCCS Centro Neurolesi “Bonino-Pulejo”, Messina, Italy; 3 Department of Social Sciences and Public Health, University of Cagliari, Cagliari, Italy; 4 Mailma School of Public Health, Columbia University, New York, USA; 5 Soziale Arbeit und Gesundheit, University of Applied Sciences Emden/Leer, Emden, Germany

**Keywords:** City, Elderly, Quality of Life, Rural Area, Urban Area, Violence

## Abstract

**Introduction:**

Two main demographic phenomena have substantially changed the condition of elderly: the growth of the urban population and the increase in longevity.

**Objective:**

The aim of the present review is to investigate how the elderly living in the cities perceive the sense of insecurity compared to those who reside in rural areas, and their Quality of Life (QoL).

**Method:**

Studies published from January 2011 to August 2017 were identified on Google and PubMed combining the following terms: “elderly urban/rural QoL” or “old age urban/rural QoL”.

**Results:**

We found 18 different papers published. However, there was only one study on how the elderly perceive the violence in the city. Studies on quality of life were not univocal. Studies on depressive disorders in old age were most homogeneous showing a condition worsening in the cities. A study on the perception of violence in US showed in residents of cities and neighborhoods with the entertainment arena and casinos an increase of criminality perception. In contrast, the crime decreased in both above-mentioned neighborhoods.

**Conclusion:**

The condition of elderly in the cities is changed considerably in the recent years. It is estimated that this trend will increase in the coming years. We do not know how older people are experiencing these changes and how they perceive the persistence of violence in the cities. Future researches must satisfy this need by addressing the issue with appropriate methodological tools. This is a public health priority.

## 1 INTRODUCTION

Two main demographic phenomena have substantially changed the condition of elderly according to the living in the city or in rural villages nowadays. First one is the growth of the urban population. This trend has been so impetuous that was impossible to adapt the architecture and the urban designs, at least in most cities, to the new demographic emergency: in 1950 30% of the world population lived in urban areas, it has increased to 54% in 2014 and it is expected to reach 66% in 2050 [1].

Although most of the new arrivals are made up of young people [1], the elderly who remain in the city, especially those with poor income in the suburban areas with maximum growth, live in a world that is quickly changing and it seems that not always it is changing in a size adapted for them [2]. In contrast, some rural areas are becoming deprived of young people. For this reason, the elderly lose their emotional support and the help in everyday life that a traditional extended family offered in the past [3].

The second phenomenon is the increase in longevity. In the world people aged over 60 were 607 million in 2000, then they became 901 million in 2015, and they will be around 1.4 billion in 2030. In 2050, the population of older persons will become double than of 2015, reaching around 2.1 billion. The people aged over 80, is growing even faster [4]. Aging is marked by loss of autonomy and the rise of chronic diseases. Thus, DALYs (Disability-Adjusted Life Years) will increase proportionally, with a dramatic burden on social and health costs [5, 6].

In a city that seems to grow too fast, why you can't plan the necessary infrastructures? How can you provide the welfare and support to the weakest?

In a field where the simplest solutions, as systematic institutionalization of elderly people with low autonomy, are certainly not the best solutions for their quality life (QoL), it isn't certain that they are even the least costly solutions.

The above mentioned phenomena are of such an epochal relevance that their study and the implementation of any preventative action on their possible health and mental health consequences should be a priority in the fields of public health and global mental health. The aim of present review is to investigate how the elderly living in the cities perceive the sense of insecurity compared to those who reside in rural areas, and to study relation between this perception and QoL.

## 2 METHODS

We carried out a systematic research on studies that compared “elderly urban/rural quality of life” or “old age urban/rural quality of life” (as key words used in Google and PubMed for search) from January 2011 to August 2017.

## 3 RESULTS

We found 14 different studies with 18 papers published (Table **1**). Results were found in Zhejiang Province, China [7]; Wardha district, India [8]; US-National Health and Nutrition Examination Survey [9]; Hai Duong Province, Vietnam [10]; Italy [11]; Japan [12]; Siliguri, India [13]; Uberaba-Minas Gerais, Brazil [14-17]; Poland [18, 19]; Serbia [20]; Pimenta Bueno (RO), Brazil [21]; Greece [22]; Sweden [23]; Kottayam District, India [24]; Abre Campo, Belo Horizonte, Brazil [25].

### 3.1 Do the Elderly Live well in the Cities? Under which Conditions do the Elderly Live Poorly in the City?

In this framework of transition is impossible to say if the elderly live well in the cities. This question is probably not useful in this general proposition. There are no systematic comparisons between the conditions of elderly in cities and rural areas of same regions. The great mobility of populations in itself can introduce bias in cross-sectional studies.

The results of these researches are not univocal. Two extensive researches in Italy [11] and Brazil [14] showed the perception of a worse QoL in urban elderly. A vast search in Sweden found no differences [23]. Some researchers found discordant results between cities and countryside in elderly who had different perception of QoL. Researches in Japan [12] and U.S [9] found a better QoL in the cities; a study on a little Serbian sample [20] found no differences.

The limits are obvious. They are induced by transversal designs, by the different social e cultural conditions and the level of development of the area investigated in the city and countryside; by the different percentage of institutionalized elders (in cities and rural areas) who did not participate in the “community” household studies; by the cultural factors as determinants of adherence; by different adherence rates, usually lower in cities and often lower in the more disadvantaged neighborhoods; by the difference of accessibility to a home fixed telephone when searches were conducted on samples selected on telephone directories, or even conducted by telephone.

Prospective studies are at the beginning. A recent cohort survey conducted in New Zealand highlighted how the QoL and depressive symptoms were related to: house ownership, economic living standard, urban versus rural residence, length of residence, ethnicity, age, and household composition [26].

Studies on depressive and mood disorders in old age are most homogeneous concerning a condition worsening of elderly in the cities, at least in western societies as found by a German survey [27], by the whole results of the World Health Survey in Sao Paolo, Brazil [28] and by the results of urban versus rural elderly people from Sardinia, Italy [29].

Conversely, studies in China [30-32] and Vietnam [33] on whole population found in the recent past a different trend with risk for depression of all the people (and partially of elderly) of rural areas against those of urban ones. Nevertheless, a recent study has examined the relationship between different household registration statuses and depressive disorders. In 7409 older adults in China, authors found temporary rural-to-urban migrant people at high risk for depression, permanent rural-to-urban migrant people at risk for depression, and urban local citizen not at risk. In addition, elderly with changes in their residential status before age 16 were more likely at risk of depressive symptoms [34].

The results of such study therefore suggest that is likely a shift of people with high frequency of depression from a brief migratory experience in cities towards the return to villages. A phenomenon that has already been hypothesized in a study repeated over time in Sardinia [3, 35, 36]. Obviously, the cross sectional model allows to formulate causative hypotheses not to test them.

In general, all these researches may not be able to answer to questions but may suggest hypothesis on risk and protecting factors to be tested in well-equipped studies and with longitudinal designs [37]. For example, a good protective factor could be the fact of being “at home” and “in family” for a not totally autonomous elder, as reported by study in which it seems that phenomenon is more widespread in rural than in urban areas [11].

Ultimately, this step is necessary to reflect on what aspects need to be investigate and on which factors is possible to create a protective role.

### 3.2 City, Slums and Violence in the Perspective of the Elderly People: Why are Studies Missing?

About one third of the population of cities, and ever more in the megacities, lives in disadvantaged circumstances that are described as “slums”. Due to the impetuous flux of people to the cities this fraction is predicted to grow tremendously in the next years [38].

A stimulating environment could offer many opportunities to change their lives for people becoming to conditions of extreme poverty or war. The theory of goal striving stress [39] has been explicated as an apparently not favorable condition, for those who live in a site for generations, but could appear as offering riches and opportunities for people arriving from areas of greater deprivation.

But in a changing world, the adaptation has difficulties ever for the newcomers youngest, even more for older people who come from a rural world that has certainly slower pace, and also for who, on the contrary, already lived in suburban neighborhoods that are changing totally their own physiognomy.

On the other hand, it is incontestable that some, not all, cities have become the most dangerous places in the world. For example the U.S. homicide rate declined by nearly half (49%), from 9.3 homicides per 100,000 U.S. residents in 1992 to 4.7 in 2011, falling to the lowest level since 1963. However, some cities maintained rates of homicide over 50 per 100,000 (Saint Louis and Baltimore) or over 40 (Detroit and New Orleans). The same trend was found in Europe that is known as “the safest continent in the world”. However, also some European cities, such as Rotterdam in Holland or Lodz in Poland’s, show rate of homicides really over the average of the continent or the respective countries [40].

However, there is a total impressive lack of studies of how elderly live violence in the city and what consequences it has on their own life and autonomy.

It is paradoxical that some indications come from a study not specifically conducted on the elderly and not conducted in particularly deprived areas [41]. This study was carried out with the aim “to assess the effect of community economic development efforts on neighborhood residents’ perceptions on violence, safety, and economic benefits”. Six Pittsburgh neighborhoods, in which casinos and sport arenas were built with the objective of increase the economy, were involved. Thus, the study was “focused on evaluating interventions and policies designed to change community economic conditions or characteristics of the physical environment” [41]. Due to the choice of the sample (from telephone directories) and the method of conduction (by telephone), the study actually selected a sample composed in most of elders: actually it was a sample of over 60 years of average age and a proportion of inactive people at work over 60% compared to about 7% of the reference population (probably due to the high rate of retirement due to the large amount of old people). Both residents in the neighborhood with the entertainment arena and in the neighborhood with the casino felt there was an increase of criminality. In contrast, the crime decreased in both neighborhoods.

This strange result is only understandable if one takes into account that a sample of predominantly old people had difficulty adjusting to a change. Although, this change meant an increase in the number of jobs and economic benefits, the respondents themselves found it.

If this happens in suburbs rich and safe enough, it can only hypothesize what is perceived on security of elderly in depressed suburbs, with high rates of crime and an impressive increase in population from new arrivals with different cultures. This is a lack of knowledge that needs to be overcome.

## CONCLUSION

The condition of elderly in the cities is changed considerably in the recent years. This trend is estimated not to diminish but will increase in the coming years.

However, we do not know how older people are experiencing these changes, how they perceive the persistence of violence in cities and how these phenomena impact their daily lives.

Future researches must necessarily satisfy this need by addressing the issue with appropriate methodological tools.

Given the proportions of the phenomenon of migration to the cities and the increase in life expectancy, this is a public health priority.

## Figures and Tables

**Table 1:** Urban/Rural comparison on elderly quality of life in cities and rural areas.

**Study**	**Country**	**Instruments**	**Sample**	**Main results**
Urosevic *et al*. 2015	Serbia	European Euro-QoL questionnaire (EQ-5D-3L)	100 elders	Poor score for anxiety/depression in rural. No differences in QoL.
Baernholdt *et al*. 2012	USA	Health related QoL (HQOL)	911 elders	People in rural had lower HQOL than people in adjacent and urban areas.
Dos Santos *et al*. 2014	Brazil	World Health Organization Quality of Life-BREF (WHOQOL-BREF) and World Health Organization Quality of Life Assessment for Older Adults (WHOQOL-OLD).	2142 elderly in urban area and other 850 in rural area.	Rural elders had scores significantly higher than the urban area in the domains of physical, psychological, and social relations in the WHOQOL-BREF, and in the facets of autonomy, past, present and future activities, social participation and intimacy of the WHOQOL-OLD.
Zhou *et al* 2011	China	Short Form Health Survey (SF-36)	2441 rural and 2554 urban participants	Scores of SF-36 in the rural population were significantly lower than those in the urban population except general health.
Werling *et al* 2016	Sweden	Survey on QoL.	587 respondents in urban areas and 123 in the rural areas.	No differences on QoL between the urban and rural elderly.
Usha & Lalitha, 2017	India	WHO QOL-BREF-26	830 rural and 120 urban elderly	The urban elders showed better QOL than those rural.
Barbosa *et al*. 2015	Brazil	WHOQOL BREF and IPAQ Long Version Questionnaire	20 residents of urban areas and 20 residents of rural areas	No differences between the rural and urban areas for QOL or PAL.
Mudey *et al*. 2011	India	WHO-QOL BREF	800 elderly subjects selected from urban (n= 400) and rural (n= 400) areas.	Elders living in the urban community reported significant lower level of QoL in the domains of physical and psychological than the rural elderly populations. The rural elderly population reported significant lower level of QoL in the domain of social relation and environmental than urban population.
Pappaionou *et al*. 2015	Greece	The Oral Health Impact Profile in its short form (OHIP-14) and Oral Health Related Quality of Life (OHRQoL).	501 elderly	The overall level of OHRQoL was better in urban than in rural elderly.
Huong, *et al*. 2012	Vietnam	Qualitative methods to explore and compare the dimensions of QoL for urban and rural dwelling elderly Vietnamese.	Sample size not found.	For elderly participants in both urban and rural areas, physical health, social relations, finances and economics, the physical and social environment, and psychological health were reported as important. Rural participants also identified religious practice as an important dimension of QoL.
Sewo Sampaio *et al*. 2013	Japan	WHOQOL-BREF and WHOQOL-OLD	830 community-dwelling older adults.	Participants living in the urban area had higher QOL scores than those living in the rural area.
Akbar *et al*. 2013	India	WHOQOL-BREF	263 geriatric subjects enrolled 172 were from rural area and 91 were from urban areas.	The urban geriatric population had a higher score compared to rural population for physical, social relationship and environmental domains. Rural subjects scored higher for Perceived Overall Quality of Life and Perceived Overall Health Status, but the difference was not found to be statistically significant.
Kostka *et al*. 2014	Poland	Body mass index (BMI), calf circumference (CC) and the Mini Nutritional Assessment (MNA) related to QoL by EuroQoL 5D questionnaire	1003 community-dwelling subjects from the urban environment, 890 subjects from the rural environment and 879 subjects from an institutional environment (nursing homes)	Nutrition status indices (BMI, CC and MNA) were generally higher in the urban than in the rural environment and clearly worse in institutionalized elderly. In both community-dwelling groups, BMI and CC were negatively related to several EuroQoL scores.
Amorim *et al*. 2017	Brazil	279 retired individuals	Scales of happiness, social support, diversity of activities, and issues about satisfaction with health and economic situation.	Retirees from the urban area had a higher happiness level than retirees from the rural area.
Carta *et al*. 2012	Italy	Short Form Health Survey (SF-12)	286 male and 399 female ederly.	The urban/rural difference of mean scores of SF-12 did not achieve statistical significance in women. Men aged 65 years and older with rural residence showed higher scores than men from the same age group with urban residence.
